# Overview of the current use of levosimendan in France: a prospective observational cohort study

**DOI:** 10.1186/s13613-023-01164-3

**Published:** 2023-08-08

**Authors:** Bernard Cholley, Mirela Bojan, Benoit Guillon, Emmanuel Besnier, Mathieu Mattei, Bruno Levy, Alexandre Ouattara, Nadir Tafer, Clément Delmas, David Tonon, Bertrand Rozec, Jean-Luc Fellahi, Pascal Lim, François Labaste, François Roubille, Thibaut Caruba, Philippe Mauriat, for the ARCOTHOVA study group, Olivier Barbot, Berthomieu Laurent, Anne-Marie Besselat, Blanchart Katrien, Adrien Bougle, Pierre Bourgoin, Causeret Arnaud, Hélène Charbonneau, Mircea Cristinar, Olivier Desebbe, Veldat Eljezi, Thibaud Genet, Maxime Grenier, Pierre Grégoire Guinot, Stéphane Lebel, Yael Levy, François Lion, Jacques Mansourati, Stéphanie Marlière, Anne-Céline Martin, Alexandre Mebazaa, Usman Mohammad, Jacques Monsegu, Nicolas Nessler, Isabelle Orsel, Etienne Puymirat, Morgan Recher, Sabri Soussi, Vincent Troussard, Sabrina Uhry, Xavier Zirphile

**Affiliations:** 1 Department of Anaesthesiology and Intensive Care Medicine, Hôpital Européen Georges Pompidou, AP-HP, 75015 Paris, France; 2 Université Paris Cité, INSERM, UMR_S 1140 “Innovations Thérapeutiques en Hémostase”, 75006 Paris, France; 3 Pôle Cardiopathies Congénitales, Hôpital Marie Lannelongue, Groupe Hospitalier Paris-Saint Joseph, 92350 Le Plessis-Robinson, France; 4 Department of Cardiology, University Hospital Besancon, Besançon, France; 5 Department of Anaesthesiology and Critical Care, Normandie Univ, UNIROUEN, INSERM U1096, CHU Rouen, 76000 Rouen, France; 6 Department of Cardiology and Cardiac Surgery, CHRU de Nancy, Hôpital de Brabois, Vandoeuvre-les Nancy, France; 7 CHRU Nancy, Critical Care, CHRU de Nancy, Hôpital de Brabois, Vandoeuvre-Les Nancy, France; 8 Department of Cardiovascular Anesthesia and Critical Care, CHU Bordeaux, CHU de Bordeaux, 33000 Bordeaux, France; 9 Biology of Cardiovascular Diseases, Université de Bordeaux, INSERM, U1034, 33600 Pessac, France; 10 Cardiology Department, Rangueil University Hospital, Toulouse, France; 11 Department of Anaesthesiology and Critical Care Medicine, University Hospital Timone, AP-HM, Aix-Marseille University, 13385 Marseille CEDEX 05, France; 12 Department of Anaesthesiology and Critical Care, Institut du Thorax, Laennec Hospital, CHU de Nantes, and Nantes Université, CHU Nantes*, CNRS, INSERM, 44000 Nantes, France; 13 Department of Cardiothoracic Anaesthesiology and Critical Care, Louis Pradel University Hospital, Lyon, France; 14 Cardiology department, Henri-Mondor University Hospital, AP-HP, Créteil, France; 15 Department of Anaesthesiology and Critical Care Medicine, Rangueil University Hospital, Toulouse, France; 16 Cardiology Department INI-CRT PhyMedExp INSERM, CNRS CHU de Montpellier, Université de Montpellier, Montpellier, France; 17 Department of Pharmacy, Hôpital Européen Georges Pompidou, AP-HP, Paris, France

**Keywords:** Levosimendan, Inotropes, Heart failure, Cardiogenic shock, Repetitive infusions, VA-ECMO weaning

## Abstract

**Background:**

Following the results of randomized controlled trials on levosimendan, French health authorities requested an update of the current use and side-effects of this medication on a national scale.

**Method:**

The France-LEVO registry was a prospective observational cohort study reflecting the indications, dosing regimens, and side-effects of levosimendan, as well as patient outcomes over a year.

**Results:**

The patients included (*n* = 602) represented 29.6% of the national yearly use of levosimendan in France. They were treated for cardiogenic shock (*n* = 250, 41.5%), decompensated heart failure (*n* = 127, 21.1%), cardiac surgery-related low cardiac output prophylaxis and/or treatment (*n* = 86, 14.3%), and weaning from veno-arterial extracorporeal membrane oxygenation (*n* = 82, 13.6%). They received 0.18 ± 0.07 µg/kg/min levosimendan over 26 ± 8 h. An initial bolus was administered in 45 patients (7.5%), 103 (17.1%) received repeated infusions, and 461 (76.6%) received inotropes and or vasoactive agents concomitantly. Hypotension was reported in 218 patients (36.2%), atrial fibrillation in 85 (14.1%), and serious adverse events in 17 (2.8%). 136 patients (22.6%) died in hospital, and 26 (4.3%) during the 90-day follow-up.

**Conclusions:**

We observed that levosimendan was used in accordance with recent recommendations by French physicians. Hypotension and atrial fibrillation remained the most frequent side-effects, while serious adverse event potentially attributable to levosimendan were infrequent. The results suggest that this medication was safe and potentially associated with some benefit in the population studied.

**Supplementary Information:**

The online version contains supplementary material available at 10.1186/s13613-023-01164-3.

## Background

The calcium-sensitizing inodilator levosimendan has been in clinical use for two decades, during which it has been subject of extensive evaluation. However, after the publication of several randomized controlled trials [1–4] failing to demonstrate its effectiveness over placebo or conventional inotropes, the place of levosimendan in the therapeutic armamentarium remains questionable. Current guidelines still recommend that levosimendan be used instead of dobutamine in patients with heart failure (HF) receiving beta-blockers [5]. A recent multicentre retrospective study and meta-analyses also suggested that levosimendan could probably be administered as repetitive infusions to support patients with advanced chronic HF awaiting heart transplantation or left ventricular assist devices (LVAD) implantation, or as palliative strategy to improve quality of life and decrease hospitalization [6–8]. An expert opinion paper also suggested a potential benefit in patients with cardiogenic shock due to left or right ventricular failure, in isolated coronary artery bypass graft patients with low left ventricular ejection fraction (as preoperative infusion), in patients awaiting weaning from veno-arterial extracorporeal membrane oxygenation (VA-ECMO), and in patients with Takotsubo syndrome [9]. In parallel, an international expert panel made similar recommendations, and proposed to use levosimendan as a first-line therapy in patients with cardio-renal syndrome [10].

In France, levosimendan has been approved on October 20th, 2015 for short-term use in adults with acute decompensation of severe chronic heart failure, unresponsive to usual treatment. Shortly after, due to the lack of strong evidence supporting the benefits of this medication, French health authorities (Haute Autorité de Santé) requested a large collection of “real life” data concerning the baseline and outcome characteristics of French patients treated with levosimendan, the treatment indications and regimens, as well as a thorough review of the side-effects. Here, we report the results of the France-LEVO registry, in which data were prospectively collected over a year to address these requirements.

## Methods

The study protocol was approved by the French Haute Autorité de Santé on October 22, 2018 (#DEMSP/SEM/AA/MPi/JT/18.0226). All patients receiving levosimendan were eligible for inclusion, after a written informed consent had been signed by themselves or by a next of kin. Outcome data were collected until day-90 after hospital discharge. The French National Committee for Informatics and Liberty approved the data collection and storage for the France-LEVO registry (CNIL, authorization 2215621, October 29th, 2019), and the cohort was registered on The French Health Data Hub (IO222141202020, January 14th, 2020) and on Clinical Trials (NCT04252404, January 31st, 2020).

Previous users of levosimendan (cardiologists, intensivists and anaesthesiologists) from public and private hospitals, both from adult and paediatric units, were identified through the orders they placed with Orion Pharma, the supplier of levosimendan in France. In 2019, levosimendan had been prescribed in 75 centres: 33 university hospitals, 31 general hospitals and 11 private hospitals. All were contacted to participate in the registry as investigators. In addition, several French scientific societies were asked to send an information letter to their members, inviting them to participate in this registry (list in Additional file 1). Anonymous data were collected and stored using an electronic case report form (CleanWeb^™^).

Investigators were asked to report hypotension (defined as a reduction in mean arterial blood pressure requiring introduction of vasoconstrictors and/or reduction or discontinuation of levosimendan infusion by the attending physician), atrial fibrillation, serious adverse events (need for mechanical circulatory support, renal replacement therapy, stroke, myocardial infarction, arrhythmia, pulmonary oedema, death from any cause), as well as any adverse event that occurred following levosimendan administration. Hypotension and new onset atrial fibrillation were recorded when occurring within 6 days following levosimendan initiation, while other adverse events were recorded without time limits. Three independent experts, blinded to the results of the registry, were asked to determine if the events were potentially associated with levosimendan. According to standard terminology, the frequency of events was considered very common if incidence was > 10%, common if comprised between 1 and 10%, uncommon (0.1–1%), rare (0;01–0.1%) and very rare if < 0.01%.

### Statistical analysis

The number of patients to be included was estimated to be 600, to allow for reporting a 50% proportion with 4.5% accuracy and 20% of missing data. Continuous variables were expressed as mean and standard deviation (SD) when normally distributed, and as median and inter-quartile range (IQR) otherwise. Data were compared using Student’s *t* test, Mann–Whitney *U* test, or analysis of variance, as appropriate. Categorical variables were expressed as numbers and percentages and compared with Chi-square test, or the Fisher’s exact test, and the Freeman–Halton extension, as appropriate. Analysis was conducted in intention to treat.

All variables significantly associated with the outcome/survival variables in univariate analysis (*p* < 0.10) were included in the multivariate prediction models. Continuous variables were analysed using multivariable regression models if normally distributed, and using general additive models otherwise. Binary variables were analysed using multivariable logistic regression models. Survival analysis used Cox multivariable models. Stepwise regression was performed, with backward selection of the variables retained in the model according to the Akaike criterion.

Patients with missing values concerning outcome and survival data were not included in the respective analyses. When adjustment variables had missing values, they were imputed by the median of the observed values. Because the daily inotrope dosages were not available, an approximation of the vasoactive-inotropic score (VIS_approx_) on a given day was calculated as follows [11]: 1 for dobutamine + 100 for norepinephrine + 100 for epinephrine + 10 for milrinone. Subgroup analyses were performed for the most prevalent treatment indications.

Observed mortality rates were compared with previously reported rates in similar populations, by comparing 500-resampled bootstrapping-estimated confidence intervals. All analyses were performed using R (version 4.0.5).

## Results

Patients were included between February 3rd 2020 and January 26th, 2021. The follow-up was stopped on May 6th, 2021. Investigators (*n* = 130) from 60 hospitals agreed to participate, and 121 investigators from 52 hospitals included at least one patient.

### Patient characteristics

A total of 602 patients (57 ± 19 years old) were included, among which 36 were less than 18 years old. The patient baseline characteristics are shown in Table 1*.* According to the data provided by Orion-Pharma, the number of drug units delivered to patients included in the registry represented 29.6% of the overall number of drug units delivered in France during the same period. The reason for initial hospital admission was decompensated heart failure in 295 (49%) patients, cardiac surgery in 167 (27.7%), and other reasons in 140 (23.2%). However, at the time of levosimendan initiation (which occurred after various delays following admission), the indication to start the treatment was cardiogenic shock in 250 patients (41.5%), decompensated heart failure in 127 (21.1%), cardiac surgery-related in 86 (14.3%), weaning from VA-ECMO in 82 (13.6%), and another reason in 57 (9.5%) patients. The underlying pathologies in patients with cardiogenic shock were: decompensated heart failure (*n* = 134, 53.6%), acute myocardial ischaemia (*n* = 39, 15.6%), postoperative (*n* = 35, 14%), dilated cardiomyopathy (*n* = 19, 7.6%), arrhythmia (*n* = 7, 2.8%), myocarditis (*n* = 4, 1.6%), and other indications (*n* = 12, 4.8%). Among cardiac surgical patients, levosimendan was used postoperatively to treat low cardiac output syndrome (LCOS) in 45 (7.5%), and was used as pharmacological prophylaxis of LCOS in 41 (6.8%) patients. The cardiac surgery patients underwent isolated valve surgery (*n* = 34, 39.5%), isolated CABG (*n* = 21, 24.4%), combined CABG/valve procedures (*n* = 6, 7%), congenital cardiac procedures (*n* = 8, 9.3%), LVAD implantation (*n* = 4, 4.6%), cardiac transplantation (*n* = 3, 3.5%), aortic surgery (*n* = 3, 3.5%) and other procedures (*n* = 7, 8.1%). Additionally, 15 (2.5%) patients received levosimendan in view of weaning from dobutamine, 10 (1.7%) to treat isolated right ventricular failure, and in 25 (4.1%) patients the reason for hospital admission was elective repeated infusion in the context of chronic heart failure.

**Table 1:** Baseline characteristics of patients: entire cohort and subgroups (main treatment indications)

Patient characteristics	All patients (n = 602)	Cardiogenic shock (n = 250)	Decompensated heart failure (n = 127)	Cardiac surgery-related^a^ (n = 86)	Weaning from ECMO (n = 82)	*P*-value
Age (years)	57.5 ± 19.4	59 ± 18	57 ± 22	55 ± 22	56 ± 16	0.37
Male	445 (73.9%)	190 (76%)	98 (77.2%)	59 (68.6%)	55 (67.1%)	0.33
SOFA score at hospital admission^b^	5.5 ± 4.1	5.68 ± 4	4.67 ± 3.54	3.68 ± 3.75	8.61 ± 3.75	< 0.001
SOFA score at levosimendan initiation^c^	5.8 ± 4.4	6.11 ± 4.22	4.76 ± 4.96	3.87 ± 3.73	9.41 ± 3.48	< 0.001
Previous atrial fibrillation	150 (24.9%)	52 (20.8%)	41 (32.3%)	20 (23.2%)	16 (19.5%)	0.02
Previous amiodarone treatment	194 (32.2%)	93 (37.2%)	46 (36.2%)	10 (11.6%)	24 (29.2%)	< 0.001
Renal replacement therapy at levosimendan initiation	65 (10.8%)	21 (8.4%)	8 (6.3%)	4 (4.6%)	27 (32.9%)	< 0.001
Ventricular support at levosimendan initiation	171 (28.4%)	63 (25.2%)	11 (8.7%)	8 (9.3%)	80 (97.5%)	< 0.001
Concomitant use of inotropes^d^	396 (65.8%)	195 (78%)	56 (44.1%)	47 (54.6%)	71 (86.6%)	< 0.001

P-values compare the patient characteristics across the main indication subgroups and the other patients. Data are shown as mean ± SD, median [IQR], or as numbers (%)

*LCOS* low cardiac output syndrome

^a^Pre-cardiac surgery-related low cardiac output prophylaxis and post-cardiac surgery-related low cardiac output treatment

^b^Missing data: 21.4%

^c^Missing data: 14.1%

^d^Concomitant inotropes at least one day during the week before and/or the week after levosimendan initiation

### Treatment regimens

**Fig. 1:**
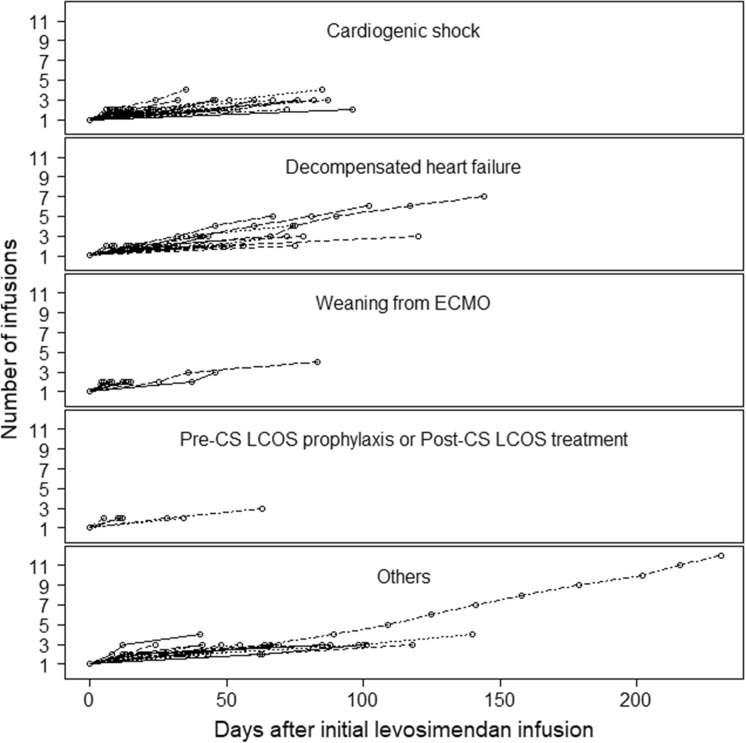
Repeated levosimendan infusions. Among “others”, 25 patients were admitted to hospital for a planned repeated infusion in the context of chronic heart failure

Among all patients, 537 (89.2%) received levosimendan in a university hospital, 19 (3.2%) in a general hospital, and 10 (1.7%) in a private hospital. On average, patients received 0.18 ± 0.07 µg/kg/min of levosimendan during 26 ± 8 h, and 45 patients (7.5%) received an initial loading dose (12 µg/kg) as a bolus. Levosimendan regimens are described in online Additional file 1: Table S1. Of all patients, 103 (17.1%) received repeated levosimendan infusions, representing a total of 268 infusions during the study period (Fig. 1). Repetitive infusions were reported in 36 (14.4%) patients with cardiogenic shock, in 25 (19.7%) patients with acute decompensated heart failure, in 15 (18.3%) patients being weaned from VA-ECMO, and in 7 (8.5%) patients undergoing cardiac surgery. The median number of infusions was 4, IQR [3, 4]; one patient received a total of 14 infusions. The average dose of each infusion was 18.1 ± 6.4 mg, and the median time interval between two infusions was 21 days, ranging between 4 to 96 days.

### Concomitant use of inotropes/vasopressors

**Fig. 2:**
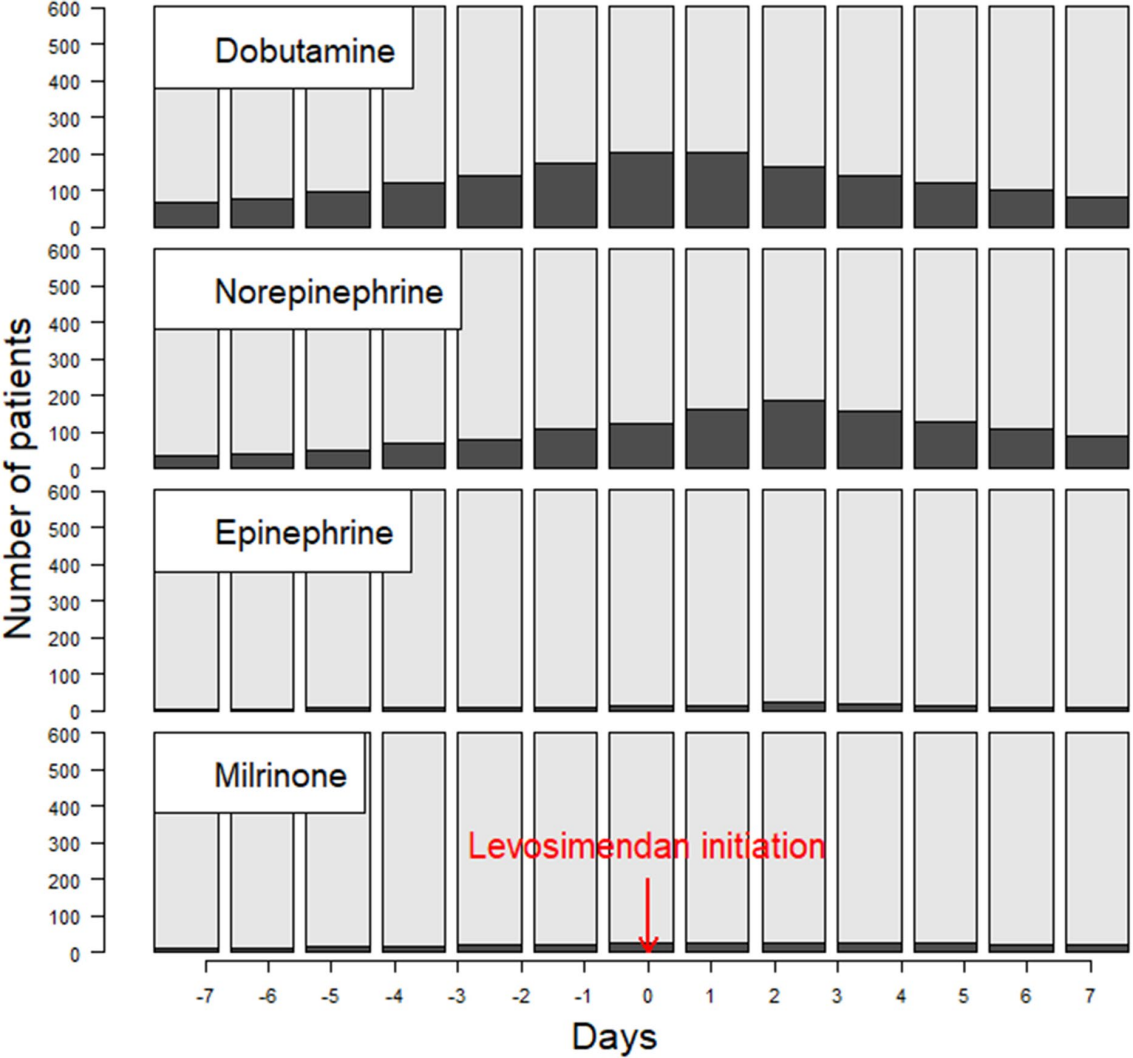
Use of inotropic agents during the week before and the week after levosimendan initiation. Day 0 corresponds to the initiation of levosimendan infusion

**Fig. 3:**
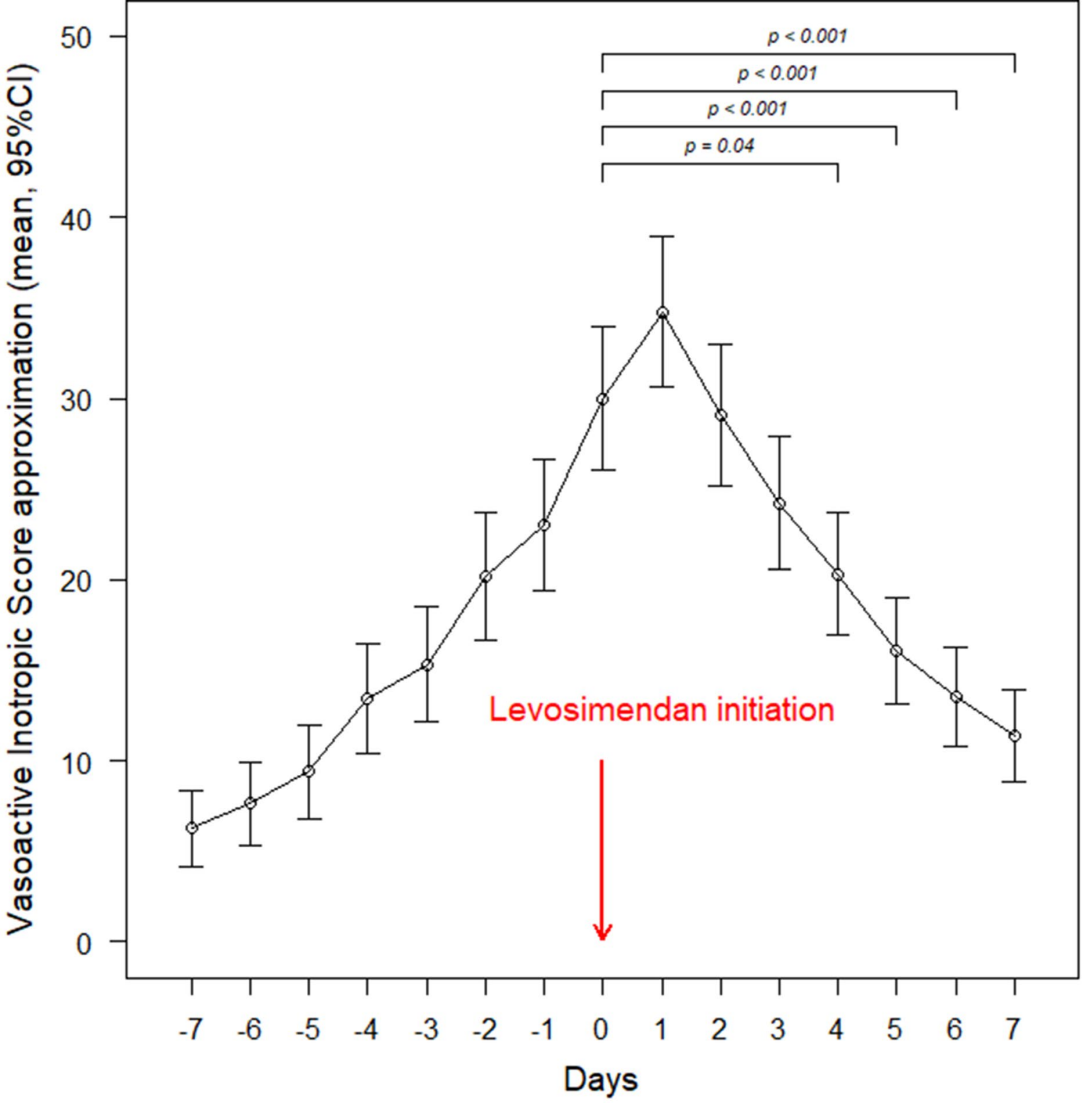
Variations in Vasoactive Inotropic Score approximation during the week before and the week after levosimendan initiation. Day 0 corresponds to the initiation of levosimendan infusion

461 (76.6%) patients received inotropes and/or vasopressors for at least one day during their hospital stay. Among them, 391 (64.9%) received the inotrope/vasopressor during the week before and/or after levosimendan infusion initiation. Dobutamine was used in 286 (47.5%) patients, norepinephrine in 247 (41.1%), milrinone in 46 (7.6%) and epinephrine in 30 (5.0%). The proportion of patients receiving these inotropes/vasopressors varied over time with respect to levosimendan initiation, as shown in Fig. 2. This proportion was different among the subgroups defined by the main indications for levosimendan in our cohort: 71 (86.6%) patients weaned from ECMO, 195 (78%) with cardiogenic shock, 47 (54.6%) of those undergoing cardiac surgery, and 56 (44.1%) patients treated for decompensated heart failure (*p* < 0.001) (Additional file 1: Fig. S1). The VIS_approx_ tended to increase on day-1 following levosimendan initiation, due to a transient increase in norepinephrine requirements. From day-2 onwards, the VIS_approx_ decreased progressively, reaching statistical significance on day-4 after initiation of levosimendan (Fig. 3 and Additional file 1: Fig. S2). This association was not explored in patients receiving prophylactic levosimendan prior to surgery (no previous inotrope), or in patients treated for “other indications” (sample size too small).

### Adverse events

218 (36.6%) patients presented at least one episode of hypotension (Table 2), and 155 (25.7%) required norepinephrine (or an increase in ongoing norepinephrine regimen) during the 6 days following levosimendan infusion and for a median of 12 [4.7–24] hours to treat this hypotension. No interruption in levosimendan infusion was reported. The patient characteristics predictive of the occurrence of hypotension are shown in Table 3.

**Table 2:** Outcome characteristics of patients: entire cohort and subgroups (main treatment indications)

Patient characteristics	All patients (*n* = 602)	Cardiogenic shock (*n* = 250)	Decompensated heart failure (*n* = 127)	Cardiac surgery-related^a^ (*n* = 86)	Weaning from ECMO (*n* = 82)	*P*-value
Hypotension after levosimendan	218 (36.2%)	108 (43.2%)	42 (33.1%)	25 (29.1%)	24 (29.3%)	0.05
Atrial fibrillation after levosimendan	85 (14.1%)	45 (18%)	13 (10.2%)	12 (13.9%)	14 (17.1%)	0.01
Serious adverse events after levosimendan	14 (2.3%)	10 (4%)	1 (0.8%)	2 (2.3%)	0	0.19
Length of Intensive Care Unit stay (days)^b^	15 [7–27]	16 [8–30]	10 [4–21]	12 [7–21]	22 [18–36]	< 0.001
Length of hospital stay (days)^b^	23.5 [13–44]	23 [13–42]	16 [10–33]	22 [14–34]	34 [18–53]	0.002
In-hospital mortality	136 (22.6%)	66 (26.4%)	21 (16.5%)	10 (11.6%)	34 (41.5%)	< 0.001
Mortality during follow-up	26 (4.3%)	9 (3.6%)	7 (5.5%)	2 (2.3%)	0	0.001

Data are shown as mean ± SD, median [IQR], or as numbers (%). Continuous variables were analysed using ANOVA, and discrete variables (contingency tables of proportions) using Fisher–Freeman–Halton test

^a^Pre-cardiac surgery-related low cardiac output prophylaxis and post-cardiac surgery-related low cardiac output treatment

^b^In survivors

**Table 3:** Independent predictors of hypotension and atrial fibrillation following levosimendan treatment (within 6 days)

Patient characteristics	Hypotension		Atrial fibrillation	
	Odds ratio	95% confidence interval	Odds ratio	95% confidence interval
Age (10-year increment)	1.03	1.006–1.05	1.03	1.02–1.05
Renal replacement therapy at the start of the treatment	1.22	1.08–1.38		NS
SOFA score at the start of the treatment		NS	1.008	1.0005–1.01
Levosimendan infusion rate (0.1 µg/kg/min increment)		NS	1.06	1.01–1.10
Hypotension following treatment	–	–	1.07	1.01–1.13
Atrial fibrillation following treatment	1.18	1.05–1.31	–	–

*NS* non–significant

New onset atrial fibrillation was observed in 85 (14.1%) patients during the 6 days following levosimendan infusion (Table 1). To manage these arrhythmias, 69 (11.5%) patients required additional amiodarone, 20 (3.3%) a beta blocker, and 25 (4.2%) an electrical cardioversion. Thirty (5%) patients had persistent atrial fibrillation after levosimendan treatment. Patient characteristics predictive of the occurrence of atrial fibrillation are shown in Table 3.

An exhaustive list of all serious adverse events reported by the investigators is shown in Additional file 1: Table S2. Overall, 17 serious adverse events were reported in 14 (2.3%) patients, and were considered to be potentially related to levosimendan by the independent expert group: 10 (1.7%) patients had arrhythmia other than atrial fibrillation occurring 2.5 [0–6] days after treatment initiation, 5 (0.8%) patients had a stroke occurring at day-2, day-3 and day-15 (2 missing values), 1 (0.2%) patient had mesenteric ischaemia at day-2 and another had myocardial ischaemia at day-2. No predictive factor for serious adverse events was identified.

### Outcome characteristics

**Fig. 4:**
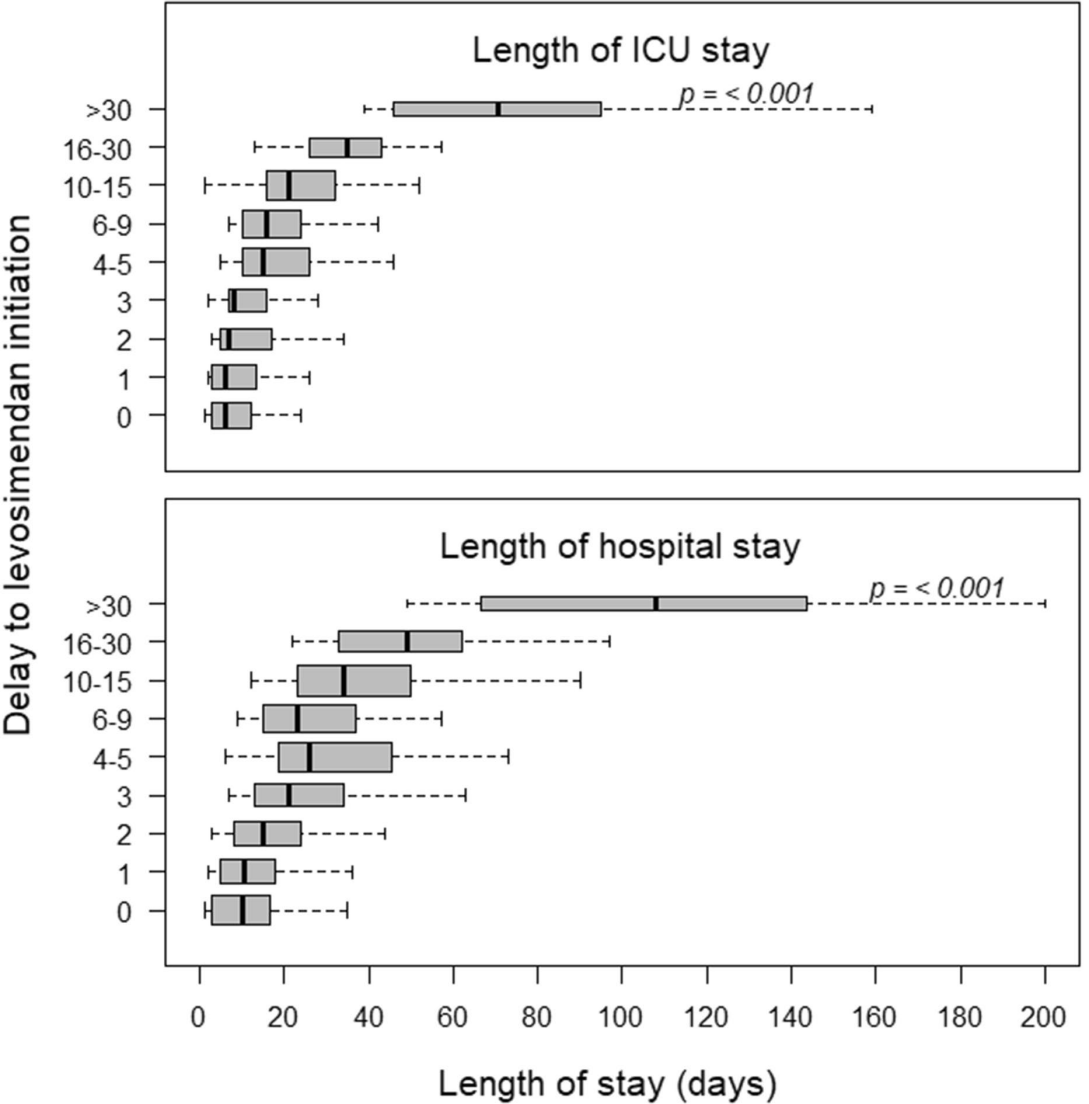
Relation between the delay to levosimendan treatment with respect to ICU admission and the duration of ICU/hospital stay. Analyses were adjusted for the variables shown in Table 4

A total of 136 patients (22.6%) died in hospital, 440 were discharged, and 26 were still in hospital at the end of the follow-up period. Another 26 patients (4.3%) died after hospital discharge, before day-90. The outcome characteristics of all patients are shown in Table 2. An earlier levosimendan initiation with respect to ICU admission was associated with shorter ICU and hospital stays (Fig. 4), a finding which was confirmed by multivariable analysis (Table 4). This was also observed in each of the four subgroups (Additional file 1: Fig. S3). However, in the “weaning from VA-ECMO” subgroup, a shorter delay to the treatment with levosimendan was not significantly associated with a shorter duration of ECMO (regression coefficient 0.18 ± 0.06, *p* = 0.08).

**Table 4:** Multivariable analysis of outcome variables

Patient characteristics	Length of ICU stay^a^		Length of hospital stay^b^		In-hospital mortality	
	Regression coefficient	*P-*value	Regression coefficient	*P-*value	Odds ratio	95% CI
Age (10-year increment)	1.41 ± 0.43	0.001	2.14 ± 0.91	0.02	1.03	1.01–1.04
SOFA score at start of levosimendan treatment^c^	0.53 ± 0.19	0.007	1.04 ± 0.38	0.006	1.01	1.004–1.02
Delay to levosimendan treatment (days)	1.08 ± 0.05	< 0.001	1.39 ± 0.11	< 0.001		NS
Dialysis prior to levosimendan treatment	11.48 ± 3.10	< 0.001		NS	1.29	1.16–1.44
Ventricular assistance prior to levosimendan treatment	4.91 ± 2.28	0.03		NS	1.13	1.05–1.23
Hypotension during levosimendan treatment		NS		NS	1.12	1.07–1.22
Inotropes at least one day during the week before and/or after starting levosimendan treatment		NS		NS	1.13	1.06–1.20
Total dose of levosimendan (mg)		NS		NS	0.99	0.98–0.99

*NS* non–significant

^a^7% missing values

^b^4.3% missing values

^c^14.1% missing values imputed by the median of the observed values, which was 5. Patients less than 18 years of age were not included in this analysis

A total of 166 (27.6%) patients were re-admitted to the hospital during the 90-day follow-up period. The most common reasons included cardiogenic shock with multi-organ failure in 33 (5.5%) patients, arrhythmia in 25 (4.2%), repeated levosimendan infusions in 23 (3.8%), percutaneous procedures in 22 (3.6%) and cardiac transplantation in 15 (2.5%).

The most common causes for in-hospital death were cardiogenic shock with multi-organ failure in 66 (11%) patients, septic shock (14, 2.3%), withholding of life-sustaining treatment (13, 2.2%), stroke (8, 1.3%), arrhythmia (7, 1.2%), mesenteric ischaemia (3, 0.5%), and ARDS (3, 0.5%). The most common causes of death during follow-up were cardiogenic shock with multi-organ failure (12, 2%), unknown (5, 0.8%) and septic shock (3, 0.5%). The patient characteristics independently associated with the risk of in-hospital death are presented in Table 4*.*

## Discussion

The France-LEVO registry allowed for the collection of data reflecting the indications, dosing regimens, outcomes and side-effects of levosimendan treatment in a real-life setting in France over a year. The data collected concerned 29.6% of all patients receiving levosimendan in France over that year. Levosimendan was administered mainly in university hospitals and for accepted indications. Hypotension and atrial fibrillation were the most frequently reported side-effects. Serious adverse events were reported in less than 2.5% of all patients. The observed reduction in concomitant inotropes following levosimendan initiation suggests that there was an improvement of the haemodynamic state in patients, although no objective haemodynamic measurements was available. The findings also suggest that an earlier start of levosimendan was associated with an earlier discharge from ICU and hospital.

### Cardiogenic shock

There is currently no high-quality study available to recommend levosimendan in cardiogenic shock, and the most recent meta-analysis failed to show a robust advantage over dobutamine with regard to mortality, ischaemic events, kidney injury, dysrhythmias or ICU length of stay [12]. However, the use of the conventional inotropes, i.e.: catecholamines and phosphodiesterase III inhibitors, increases the risk of mortality and major adverse events in patients with heart failure [13, 14] or postoperative cardiogenic shock [15, 16]. This is, especially the case in situations of myocardial reperfusion injury [17]. Currently, levosimendan is recommended as a rescue therapy after failure of dobutamine and before envisaging mechanical support. The ongoing LevoHeartShock trial, comparing levosimendan vs placebo on top of a conventional catecholamine inotrope strategy on a combined morbidity-mortality endpoint, has been designed to test the hypothesis of a beneficial effect of early administration of levosimendan in patients with cardiogenic shock.

One of the potential difficulties with the use of levosimendan in cardiogenic shock is the potent vasodilator effect, which may lead to increased vasopressor requirements. This is illustrated in Additional file 1: Fig. S1, showing a peak of norepinephrine usage at day-1 and 2 after levosimendan initiation in patients with cardiogenic shock.

### Decompensated heart failure

Subgroup analyses of the LIDO and SURVIVE trials suggested a haemodynamic benefit when levosimendan was used in patients treated with beta-blockers with acute decompensated heart failure [18, 19]. Based on these results, levosimendan has become the drug of choice in such patients [5]. More than 20% of the patients included in the France-LEVO registry received levosimendan for decompensated heart failure, and, although previous medications were not recorded, beta-blockers are the first-line therapy for chronic heart failure in France. In accordance with previous findings, the present data show a significant decrease in inotropic support after levosimendan initiation (Additional file 1: Figs. S1 and S2), and they suggest an association between faster recovery and early levosimendan introduction (Additional file 1: Fig. S3).

Repetitive infusions of levosimendan are thought to offer the advantage of a prolonged beneficial effect, improving heart failure symptoms. This regimen allows for hospital discharge of patients with decompensated heart failure, dependent from continuous inotropic infusions, and may improve survival [6, 7, 20–22]. In the present cohort, 20% of all patients admitted for decompensated heart failure had repeated levosimendan infusions, and 4% were electively admitted to repeat such infusions*.*

### Weaning from VA-ECMO

Two recent meta-analyses reported an increase in the likelihood of VA-ECMO weaning, with a significantly better rate of weaning in patients with low LVEF, with cardiac surgery and cardiogenic shock [23, 24]. Two ongoing multicentre randomized controlled trials (LEVOECMO and WEANILEVO [25]) aim at comparing the effect of levosimendan *vs* placebo on the likelihood of successful ECMO weaning in patients treated with VA-ECMO for cardiogenic shock and decompensated heart failure, respectively. In the present cohort, the patients with VA-ECMO represented the subgroup in which the use of classical inotropes was the most prevalent (> 85%), and for which the start of levosimendan resulted in the fastest and most important reduction in inotrope use (Additional file 1: Figs. S1 and S2). Early introduction of levosimendan was not associated with a faster weaning from VA-ECMO, but we observed shorter times to discharge (Additional file 1: Fig. S3).

### Cardiac surgery

In cardiac surgical patients with postoperative LCOS, we observed a decrease in the use of inotropes shortly after levosimendan initiation (Additional file 1: Figs. S1 and S2). There was also an association between early introduction of levosimendan and shorter ICU and hospital stays (Additional file 1: Fig. S3). Such positive effects were not observed in the CHEETAH trial, in which the postoperative use of levosimendan in patients with LCOS had no impact on the duration of mechanical ventilation, length of stay, and 30-day mortality compared to placebo [3]. Of note, higher levosimendan dosages were used here, i.e. roughly the double of the regimen used in the aforementioned trial. A small subgroup of patients received prophylactic levosimendan prior to cardiac surgery in our cohort. Not surprisingly, the present data do not suggest any impact of levosimendan on outcome.

### Tolerance

A recent meta-analysis including 16 studies found no evidence that levosimendan produces vasopressor-resistant vasoplegic syndrome [26]. Our results are consistent with this finding, since only 25.7% of all patients required addition of norepinephrine for a median period of 12 h. The prevalence of hypotension in our cohort was 36.2% (very common), a figure that is less than the 50% observed in the REVIVE II trial [27] despite the fact that we included all hypotensive episode occurring up to 6 days after levosimendan initiation. Such a long delay may have resulted in counting hypotensive episodes that were not directly related to levosimendan. Hypotension dissipated on average 12 h after the discontinuation of the drug*.* Another meta-analysis of 25 trials including 5349 patients with heart failure reported a 33% increase in the risk of hypotension when compared with placebo or dobutamine [28]. Atrial fibrillation was detected in only 14.1% of our patients, but figures as high as 50% have been reported previously [1].

Although less than 2.5% of patients presented serious adverse events that were adjudicated as potentially related to the use of levosimendan, this means that adverse events were common. The independent experts considered all ischaemic events occurring within 1 week after levosimendan initiation, in patients with no concomitant mechanical circulatory support devices, as possibly related to the use of levosimendan (Additional file 1: Table S2). These included stroke, myocardial and mesenteric ischaemia. The causative role of levosimendan is, nevertheless, hypothetical.

### Outcomes

We observed that an earlier start of levosimendan with respect to ICU admission was associated with a shorter time to ICU and hospital discharge. Together with the significant decrease in the use of inotropes following treatment initiation, these data suggest that levosimendan may be associated with beneficial effects. Moreover, mortality rates adjusted to SOFA score were lower in our cohort in comparison to previous cohorts of similar patients not receiving levosimendan (Additional file 1: Table S3).

### Limitations

The present data are observational, therefore all the associations reported here need to be confirmed externally. Although the multicentre prospective collection of the data is a major strength of the France-LEVO registry, the present findings mostly reflect the use of levosimendan in French university hospitals. We consider that information concerning almost 30% of all prescriptions nationwide during a year provides a very relevant sample of the yearly prescriptions and allows a good description of the use of levosimendan in France over the period covered. The number of items collected had to be limited in order to encourage investigators to participate. Therefore, some baseline patient characteristics, such as previous medications and LVEF before the start of levosimendan treatment, were not obtained. No objective haemodynamic assessment other than the inotrope use was available either, and only an approximation of the vasoactive-inotropic score could be calculated. Although hypotension was reported, no arterial pressure measurement or duration of decrease in arterial pressure was available in the registry. The interpretation of the association between the use of levosimendan and the occurrence of serious adverse events, although entrusted to independent experts, could have been subjective as well.

## Conclusion

These data reflect the “real life” use of levosimendan over a 1-year period in France. The cohort provides a representative sample of the prescriptions of levosimendan nationwide (one-third). Indications were in accordance with recent expert recommendations. The results suggest that this medication was safe and potentially associated with a decrease in the use of conventional inotropes after 24 h. New randomized controlled trials will be needed to confirm any potential improvement in outcome in specific patient populations.

### Supplementary Information

**Additional file 1: Table S1.** Levosimendan regimen overall and in the main indication subgroups. **Table S2.** All serious adverse events and events adjudicated by an independent expert panel. **Table S3.** Comparison of the mortality rates observed per SOFA score category in the France-LEVO cohort with mortality rates reported in previous cohorts. **Figure S1.** Use of inotropic agents during the week before and the week after levosimendan initiation according to the main treatment indication subgroups. **Figure S2.** Variations in Vasoactive Inotropic Score approximation during the week before and the week after levosimendan initiation according to the main treatment indication subgroups. **Figure S3.** Relation between the delay to levosimendan treatment and the duration of ICU / hospital stay according to the main treatment indication subgroups.

## Data Availability

The datasets used and/or analysed during the current study are available from the corresponding author on reasonable request.

## Abbreviations

HF: Heart failure
VA-ECMO: Veno-arterial extracorporeal membrane oxygenation
LVAD: Left ventricular assist device
LVEF: Left ventricular ejection fraction
LCOS: Low cardiac output syndrome
SD: Standard deviation
IQR: Interquartile range
VIS_approx_: Vasoactive inotropic score approximative
