# Correction: Overview of the current use of levosimendan in France: a prospective observational cohort study

**DOI:** 10.1186/s13613-023-01193-y

**Published:** 2023-09-29

**Authors:** Bernard Cholley, Mirela Bojan, Benoit Guillon, Emmanuel Besnier, Mathieu Mattei, Bruno Levy, Alexandre Ouattara, Nadir Tafer, Clément Delmas, David Tonon, Bertrand Rozec, Jean-Luc Fellahi, Pascal Lim, François Labaste, François Roubille, Thibaut Caruba, Philippe Mauriat, for the ARCOTHOVA study group, Olivier Barbot, Berthomieu Laurent, Anne-Marie Besselat, Blanchart Katrien, Adrien Bougle, Pierre Bourgoin, Causeret Arnaud, Hélène Charbonneau, Mircea Cristinar, Olivier Desebbe, Veldat Eljezi, Thibaud Genet, Maxime Grenier, Pierre Grégoire Guinot, Stéphane Lebel, Yael Levy, François Lion, Jacques Mansourati, Stéphanie Marlière, Anne-Céline Martin, Alexandre Mebazaa, Usman Mohammad, Jacques Monsegu, Nicolas Nesseler, Isabelle Orsel, Etienne Puymirat, Morgan Recher, Sabri Soussi, Vincent Troussard, Sabrina Uhry, Xavier Zirphile

**Affiliations:** 1 Department of Anaesthesiology and Intensive Care Medicine, Hôpital Européen Georges Pompidou, AP-HP, 75015 Paris, France; 2 Université Paris Cité, INSERM, UMR_S 1140 “Innovations Thérapeutiques en Hémostase”, 75006 Paris, France; 3 Pôle Cardiopathies Congénitales, Hôpital Marie Lannelongue, Groupe Hospitalier Paris-Saint Joseph, 92350 Le Plessis-Robinson, France; 4 Department of Cardiology, University Hospital Besancon, Besançon, France; 5 Department of Anaesthesiology and Critical Care, Normandie Univ, UNIROUEN, INSERM U1096, CHU Rouen, 76000 Rouen, France; 6 Department of Cardiology and Cardiac Surgery, CHRU de Nancy, Hôpital de Brabois, Vandoeuvre-les Nancy, France; 7 CHRU Nancy, Critical Care, CHRU de Nancy, Hôpital de Brabois, Vandoeuvre-Les Nancy, France; 8 Department of Cardiovascular Anesthesia and Critical Care, CHU Bordeaux, CHU de Bordeaux, 33000 Bordeaux, France; 9 Biology of Cardiovascular Diseases, Université de Bordeaux, INSERM, U1034, 33600 Pessac, France; 10 Cardiology Department, Rangueil University Hospital, Toulouse, France; 11 Department of Anaesthesiology and Critical Care Medicine, University Hospital Timone, AP-HM, Aix-Marseille University, 13385 Marseille CEDEX 05, France; 12 Department of Anaesthesiology and Critical Care, Institut du Thorax, Laennec Hospital, CHU de Nantes, and Nantes Université, CHU Nantes*, CNRS, INSERM, 44000 Nantes, France; 13 Department of Cardiothoracic Anaesthesiology and Critical Care, Louis Pradel University Hospital, Lyon, France; 14 Cardiology department, Henri-Mondor University Hospital, AP-HP, Créteil, France; 15 Department of Anaesthesiology and Critical Care Medicine, Rangueil University Hospital, Toulouse, France; 16 Cardiology Department INI-CRT PhyMedExp INSERM, CNRS CHU de Montpellier, Université de Montpellier, Montpellier, France; 17 Department of Pharmacy, Hôpital Européen Georges Pompidou, AP-HP, Paris, France

**Correction: Annals of Intensive Care (2023) 13:69** 10.1186/s13613-023-01164-3

In the original publication of the article [1], ARCOTHOVA study group Collaborators’ member name Nicolas Nesseler was incorrectly written as Nicolas Nessler. The original article has been corrected.
