# Supplementary material for: Overview of the current use of levosimendan in France: a prospective observational cohort study
Source: Ann Intensive Care. 2023 Aug 8;13:69. doi: 10.1186/s13613-023-01164-3 (PMC10409690; doi:10.1186/s13613-023-01164-3)
Supplement: Supplementary file 1 — Additional file 1: Table S1. Levosimendan regimen overall and in the main indication subgroups. Table S2. All serious adverse events and events adjudicated by an independent expert panel. Table S3. Comparison of the mortality rates observed per SOFA score category in the France-LEVO cohort with mortality rates reported in previous cohorts. Figure S1. Use of inotropic agents during the week before and the week after levosimendan initiation according to the main treatment indication subgroups. Figure S2. Variations in Vasoactive Inotropic Score approximation during the week before and the week after levosimendan initiation according to the main treatment indication subgroups. Figure S3. Relation between the delay to levosimendan treatment and the duration of ICU / hospital stay according to the main treatment indication subgroups. [file 13613_2023_1164_MOESM1_ESM.docx]

**Additional file**

**List of Collaborators page 2-3**

**List of Scientific Societies that participated in investigator recruitment page 4**

**Table S1 page 5**

Levosimendan regimen overall and in the main indication subgroups

**Table S2 page 6**

Serious adverse events

**Table S3 : page 7**

Comparison of the mortality rates observed per SOFA score category in the France-LEVO cohort with mortality rates reported in previous cohorts

**Figure S1 page 8**

Use of inotropic agents during the week before and the week after levosimendan initiation according to the main treatment indication subgroups.

**Figure S2 page 9**

Variations in Vasoactive Inotropic Score approximation during the week before and the week after levosimendan initiation according to the main treatment indication subgroups.

**Figure S3 page 10**

Relation between the delay to levosimendan treatment and the duration of ICU / hospital stay according to the main treatment indication subgroups

**Collaborators** *(alphabetic order)*

**Barbot Olivier,** MD, Intensive Care Unit, hospital of Perpignan, France.

**Berthomieu Laurent**, MD, Department of Pediatric Reanimation, University Hospital of Toulouse, France.

**Besselat Anne-Marie,** MD, Cardiology Department, Centre Hospitalier Mémorial of Saint-Lô, France

**Blanchart Katrien,** MD, Department of Cardiology and Cardiac Surgery, University Hospital of Caen, France.

**Bougle Adrien,** MD, Department of Anesthesiology and Critical Care Medicine, Hôpital de La Pitié Salpêtrière, AP-HP, Paris, France.

**Bourgoin Pierre,** MD, Department of Pediatric and Neonatal Critical Care, Hôpital Femme Enfant Adolescent, CHU de Nantes, France.

**Causeret Arnaud,** MD, Department of Anesthesiology and Intensive Care Medicine, Arnaud Tzanck Institut, St Laurent du Var, France.

**Charbonneau Hélène,** MD, Department of Anesthesia and Critical Care, clinique Pasteur, Toulouse.

**Cristinar Mircea**, MD, Pôle Anesthésie Réanimation Chirurgicale, service de Réanimation Chirurgicale, Hôpitaux Universitaires de Strasbourg NHC, France.

**Desebbe Olivier,** MD, Department of Anesthesiology and Perioperative Medicine Sauvegarde Clinic, Ramsay Santé, Lyon, France.

**Eljezi Veldat,** MD, Department of Perioperative Medicine, CHU Gabriel-Montpied, Clermont-Ferrand, France.

**Genet Thibaud,** MD, Department of Cardiology, University Teaching Hospital of Trousseau and EA7505, University François Rabelais, Tours, France.

**Grenier Maxime,** MD, Department of Cardiology, hospital of Arras, France.**Pierre Guinot Grégoire,** MD, PhD, Department of Anesthesiology and Critical Care Medicine, Dijon University Hospital, France.

**Lebel Stéphane,** MD, Department of Anaesthesiology and Critical Care Medicine, University Hospital Timone, AP-HM, Marseille, France.

**Levy Yael,** MD, Neonatal and Pediatric Intensive Care Unit, Hôpital Armand-Trousseau, Paris, France. Réanimation Polyvalente, Centre Hospitalier Universitaire Félix Guyon, Saint Denis, France.

**Lion François,** MD, Department of Anesthesiology and Critical Care Medicine, CHU of Martinique, Fort-de-France, France.

**Mansourati Jacques,** MD, PhD, Department of cardiology, university hospital of Brest, France.

**Marlière Stéphanie,** MD, Department of Cardiology, Grenoble University Hospital, Grenoble, France.

**Martin, Anne-Céline,** MD, PhD, Department of Cardiology, Hôpital Européen Georges Pompidou, AP-HP, Paris, France.

**Mebazaa Alexandre,** MD, PhD, Department of Anesthesia, Burn and Critical Care, Hôpitaux Universitaires Saint Louis Lariboisière, AP-HP, Paris, France.

**Mohammad Usman,** MD, Department of Interventional Cardiology, hospital of Valenciennes, France.

**Monsegu Jacques,** MD, PhD, Department of Interventional Cardiology, Institut Cardio-Vasculaire, Groupe Hospitalier Mutualiste Grenoble, France.

**Nessler Nicolas,** MD, Department of Anesthesia and Critical Care, Pontchaillou, University Hospital of Rennes, France.

**Orsel Isabelle,** MD, Department of Anesthesiology, Academic Teaching Hospital of Limoges, France.

**Puymirat Etienne,** MD, PhD, Department of Cardiology, Hôpital Européen Georges Pompidou, AP-HP, Paris, France.

**Recher Morgan,** MD, Department of Paediatric Intensive Care Unit, Jeanne de Flandre Hospital, Lille, France.

**Soussi Sabri,** MD, PhD, Interdepartmental Division of Critical Care, Faculty of Medicine, St Michael's Hospital, Keenan Research Centre for Biomedical Science and Institute of Medical Sciences, University of Toronto, Toronto, ON, Canada

**Troussard Vincent,** MD, Cardiology Department, hospital of Lisieux, France

**Uhry Sabrina,** MD, Cardiology Department, CH de Haguenau, France.

**Zirphile Xavier,** MD, Medico-surgical department of valvulopathies and cardiomyopathies, University Hospital Center of Bordeaux, France.

**List of Scientific Societies and professional bodies that participated in investigator recruitment**

- Anesthésie-Réanimation Cœur Thorax Vaisseaux (ARCOTHOVA)
- Société de Réanimation de Langue Française (SRLF)
- Groupe Francophone de Réanimation et d’Urgence Pédiatrique (GFRUP)
- Société Française de Cardiologie (SFC), and the following sections of this society :
- Groupe Urgences et Soins Intensifs de Cardiologie (USIC)
- Groupe de Réflexion sur la Cardiologie Interventionelle (GRCI)
- Groupe Athérome et Cardiologie (GACI)

**Table S1. Levosimendan regimen overall and in the main indication subgroups**

| **Treatment characteristics** | **Overall**  **(n = 602)** | **Cardiogenic shock**  **(n = 250)** | **Decompensated heart failure**  **(n = 127)** | **Cardiac surgery related***  **(n = 86)** | **Weaning from ECMO**  **(n = 82)** | **P-value** |
| --- | --- | --- | --- | --- | --- | --- |
| Delay between admission and initiation of levosimendan (days) | 5 [2 - 11] | 5 [2 – 11] | 5 [1 – 10] | 3 [0 – 6] | 7 [4.2 – 13] | 0.03 |
| Initial bolus (12 µg/kg) | 45 (7.5%) | 23 (9.2%) | 9 (7.1%) | 4 (4.6%) | 4 (4.9%) | 0.55 |
| Infusion rate (µg/kg/min) | 0.18 ± 0.07 | 0.18 ± 0.07 | 0.18 ± 0.07 | 0.19 ± 0.04 | 0.2 ± 0.07 | 0.26 |
| Total dosage (mg) | 18.08 ± 7.07 | 18 ± 6.9 | 17.9 ± 6.8 | 17.91 ± 7.32 | 19.4 ± 6.8 | 0.40 |
| Duration of levosimendan infusion (h) | 24 [24 – 25.2] | 24 [24 – 24.5] | 24 [24 – 25] | 24.3 [24 – 27] | 24 [24 – 25.2] | 0.57 |
| Treatment interruption | 23 (0.3%) | 8 (3.2%) | 6 (4.7%) | 1 (1.2%) | 3 (3.6%) | 0.20 |
| Repeated infusions | 103 (17.1%) | 36 (14.4%) | 25 (19.7%) | 7 (8.1%) | 15 (18.3%) | <0.001 |

Data are shown as mean ± SD, median [IQR], or as number (%). Continuous variables were analyzed using ANOVA, and discrete variables (contingency tables of proportions) using Fisher-Freeman-Halton test.

*Pre-cardiac surgery-related low cardiac output prophylaxis and post-cardiac surgery-related low cardiac output treatment

**Table S2. All serious adverse events and events adjudicated by an independent expert panel**

| **Reported adverse events** | **All** | | **Adjudicated as potentially associated with levosimendan** | |
| --- | --- | --- | --- | --- |
|  | **N (%)** | **Days after levosimendan initiation** | **N (%)** | **Days after levosimendan initiation** |
| Need for mechanical ventricular support | 23 (3.8%) | 8 [0 – 188] |  |  |
| Renal replacement therapy | 23 (3.8%) | 3.5 [0 - 62] |  |  |
| Septic shock | 14 (2.3%) | 9 [1 - 32] |  |  |
| Dysrythmia (other than atrial fibrillation) requiring electrical cardioversion | 10 (1.7%) | 2.5 [0 - 6] | 10 (1.7%) | 2.5 [0 - 6] |
| Cardiogenic shock | 9 (1.5%) | 4 [0 - 151] |  |  |
| Stroke | 8 (1.3%) | 15 [2 - 27] | 5 (0.8%) | 2, 3, 15  (2 missing) |
| Mesenteric and peripheral ischemia | 5 (0.8%) | 1.5 [0 - 13] | 1 (0.2%) | 2 |
| Respiratory distress syndrome | 4 (0.7%) | 41.7 [1 - 81] |  |  |
| Pulmonary edema | 3 (0.5%) | 2 [3, 6] |  |  |
| Myocardial ischemia | 1 (0.2%) | 2 | 1 (0.2%) | 2 |
| Others | 14 (2.3%) | 6 [1 - 60] |  |  |

Data are presented as: median [range]

**Table S3. Comparison of the mortality rates observed per SOFA score category in the France-LEVO cohort with mortality rates reported in previous cohorts.**

|  | **N** | **Indication** | **SOFA score calculation** | **Mortality calculation** | **Mortality per SOFA score category** | | | |
| --- | --- | --- | --- | --- | --- | --- | --- | --- |
|  |  |  |  |  | 0-1 | 2-3 | 4-5 | >5 |
| **France-LEVO**  **cohort** | 602 | Cardiogenic shock | At the start of the levosimendan treatment | Day 30 after the start of the levosimendan treatment | 4.3%  [3.4 - 14.5] | 9.1%  [3.3 - 17.3] | 14.8%  [5.6 - 24.8] | 34.3%  [24.7 - 43.6] |
|  |  | Cardiac surgery |  |  | 6.1%  [2.6 – 15.8] | 6.2%  [4.7 – 22.2] | 11.8%  [4.5 – 35.7] | 34.3%  [22.5 – 44.8] |
| **Ferreira**  **JAMA 2001** [**^33^**](#_ENREF_33) | 352 | 55% Medical  45% Cardiac surgery | At hospital admission | In-hospital | 0 | 6.5% | 20.2% | 39.9% |
| **Elias 2020** [**^34^**](#_ENREF_34) | 3232 | Cardiogenic shock | At hospital admission | Day 30 after hospital admission | 5.5% | 10.4% | 25.4% | 47.6% |

**Figure S1. Use of inotropic agents during the week before and the week after levosimendan initiation according to the main treatment indication subgroups.**


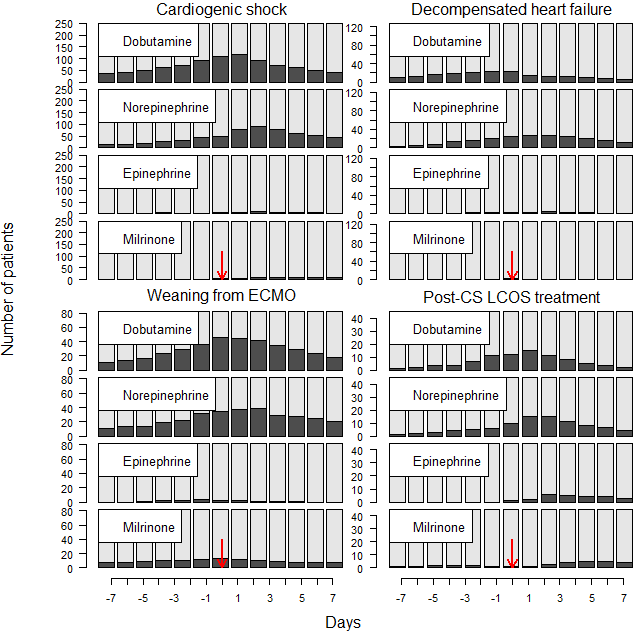


The red arrows indicate the day of levosimendan initiation. CS: cardiac surgery. ECMO: extracorporeal membrane oxygenation. LCOS: low cardiac output syndrome

**Figure S2. Variations in Vasoactive Inotropic Score approximation during the week before and the week after levosimendan initiation according to the main treatment indication subgroups.**


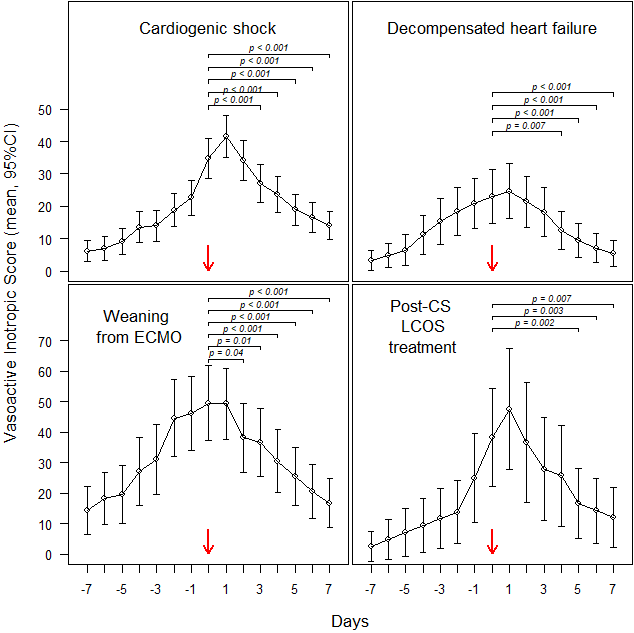


The red arrow shows the day of levosimendan initiation. CS: cardiac surgery. ECMO: extracorporeal membrane oxygenation. LCOS: low cardiac output syndrome

**Figure S3. Relation between the delay to levosimendan treatment and the duration of ICU / hospital stay according to the main treatment indication subgroups**


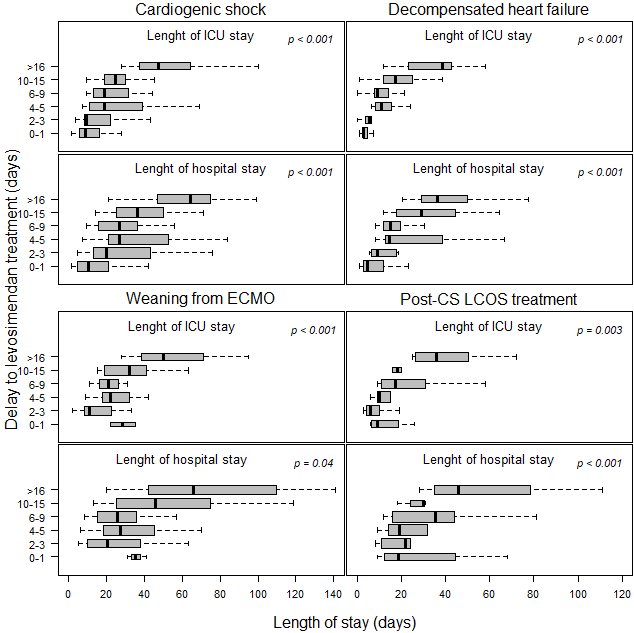


All analyses were adjusted for the variables shown in Table 4.
